# A long-term decline in downward surface solar radiation

**DOI:** 10.1093/nsr/nwaf007

**Published:** 2025-01-25

**Authors:** Fengfei Song, Yudi Mao, Shichu Liu, Lixin Wu, Lu Dong, Hui Su, Yawen Wang, Boriana Chtirkova, Peili Wu, Martin Wild

**Affiliations:** Frontiers Science Center for Deep Ocean Multispheres and Earth System and Physical Oceanography Laboratory, Ocean University of China, Qingdao 266100, China; Laoshan Laboratory, Qingdao 266237, China; Frontiers Science Center for Deep Ocean Multispheres and Earth System and Physical Oceanography Laboratory, Ocean University of China, Qingdao 266100, China; College of Oceanic and Atmospheric Sciences, Ocean University of China, Qingdao 266100, China; Frontiers Science Center for Deep Ocean Multispheres and Earth System and Physical Oceanography Laboratory, Ocean University of China, Qingdao 266100, China; College of Oceanic and Atmospheric Sciences, Ocean University of China, Qingdao 266100, China; Key Laboratory of Physical Oceanography and Frontiers Science Center for Deep Ocean Multispheres and Earth System/Academy of the Future Ocean, Ocean University of China, Qingdao 266100, China; Frontiers Science Center for Deep Ocean Multispheres and Earth System and Physical Oceanography Laboratory, Ocean University of China, Qingdao 266100, China; Laoshan Laboratory, Qingdao 266237, China; Frontiers Science Center for Deep Ocean Multispheres and Earth System and Physical Oceanography Laboratory, Ocean University of China, Qingdao 266100, China; Laoshan Laboratory, Qingdao 266237, China; Department of Civil and Environmental Engineering, The Hong Kong University of Science and Technology, Hong Kong 999077, China; College of Oceanic and Atmospheric Sciences, Ocean University of China, Qingdao 266100, China; ETH Zürich, Institute for Atmospheric and Climate Science, Zürich 8092, Switzerland; Met Office Hadley Centre, Exeter EX1 3PB, UK; ETH Zürich, Institute for Atmospheric and Climate Science, Zürich 8092, Switzerland

**Keywords:** solar radiation, CMIP6 climate models, emission scenarios, greenhouse gases, anthropogenic aerosols

## Abstract

Downward surface solar radiation (DSSR) is critical for the Earth system. It is well-known that DSSR over land has fluctuated on decadal timescales in the past. By utilizing a combination of station observations and the latest CMIP6 simulations, here we show that DSSR had a global consistent decline during 1959–2014, with comparable contributions from greenhouse gases (GHGs) and anthropogenic aerosols. The role of GHGs is even more important in the satellite period. The contribution from GHGs comes through rising temperature, which reduces the DSSR by increasing water vapor but is partly offset by reduced cloud. Future changes of DSSR are heavily dependent on climate change scenarios, which can be predicted well by global mean surface temperature (GMST) and aerosol concentrations. The sharp aerosol reduction and weak temperature rise in the SSP245/SSP126 scenarios will limit or stop the long-term decline of DSSR thus leading to a brighter future.

## Teaser

Increased greenhouse gases reduced the solar radiation reaching the Earth surface as it was absorbed more by water vapor in the air. This is the same important as the aerosols in the historical period, which reduced the solar radiation by reflecting it back into the space.

## INTRODUCTION

As a key component of Earth's surface energy budget, downward surface solar radiation (DSSR) is the ultimate energy source for life on the planet and largely determines the climatic conditions for its habitats [1–3]. As a primary factor in the energy yields of photovoltaic systems, the DSSR is becoming an attractive resource to meet the ever increasing demand for energy while reducing dependence on fossil fuels in the coming decades [4,5], in order to limit global warming below 2 or 1.5 K by 2100 [6].

Previous studies found that DSSR has exhibited significant decadal variations during the historical period based on observational datasets over land, including a widespread dimming during the 1950s–1980s and a brightening thereafter [1,7–11]. Regionally, such transitions are evident over Europe and North America [10–13]. After a similar dimming trend during the 1950s–1980s followed by a stable period in the 1990s–2000s, China has also seen a partial recovery in DSSR after 2005 [14–19]. A recovery over India has yet to be seen [20,21]. All those studies focused on land, where relatively long-period, high-quality observations are available. In contrast, knowledge of DSSR changes over the ocean is quite limited due to sparse observations. Based on one single climate model simulation driven by observed sea surface temperature (SST) and external forcings, it was reported that DSSR over the ocean has decreased in the historical period [22], but it is still unclear how realistic the model simulation is as surface energy balance has long been a challenge for generations of climate models [23–27]. Internal variability is also an important factor, even for the observed multidecadal trends [23–25,27–29], which may further increase the difficulty in examining the response of DSSR to external forcing. Hence, a global picture of long-term DSSR changes is still lacking due to the limitations in both observations and model simulations.

It is generally believed that aerosol forcing is the dominant factor for the observed decadal trends of DSSR [1,30–33]. Some previous studies, however, also argued that changes in cloud cover could be more important at shorter timescales over some regions, such as the USA [12,13] and China [12,13,34]. Water vapor is another major factor influencing DSSR by direct absorption, but it was argued as unimportant for the observed DSSR changes over land, as it cannot explain the large magnitudes of DSSR changes at station levels [1,8,10,34,35]. However, the potential importance of water vapor over the ocean and its integral effect on global DSSR changes remain unexplored. Based on one single climate model simulation, Ref. [36] suggested that water vapor can be important for future changes in DSSR. It is intriguing to note that the climate model used in Ref. [36] was one of the best among CMIP5 models in capturing the water vapor absorption of solar radiation which is generally underestimated in most models [37]. The latest evaluation of CMIP6 models against CMIP5 from the same modelling center has found that the underestimation in water vapor absorption of solar radiation has been markedly reduced by 44.9% (Fig. S1). This is also consistent with the recent argument that the latest CMIP6 models are the first generation of climate models that largely remediate the long-standing model biases in DSSR [38]. Hence, it becomes possible and urgent to examine past and future changes in DSSR at a global scale based on CMIP6 models.

By combining the Global Energy Balance Archive (GEBA) [39] station data and multiple CMIP6 model simulations [40], we have found a consistent long-term decline in global DSSR during the period 1959–2014. Analyses of model simulations driven by individual forcings show that aerosols and greenhouse gases (GHGs) have comparable contributions to the historical decline. The GHG contributions derive from increased water vapor, which is an important shortwave absorber. To our knowledge, it is the first study that highlights the role of water vapor in past DSSR changes. Although the water vapor and aerosols work together in the historical period, they would compete to determine the future projections of DSSR changes as aerosols would turn from increase to decrease in the future. The increase in water vapor is mainly controlled by the global mean surface temperature (GMST; which is tied to climate sensitivity in a particular model). For given GMST and aerosol concentrations, a prediction model is constructed to estimate the past and future DSSR changes with satisfactory results across different historical periods and multiple future scenarios. This prediction model can also be used to aid the development of future scenarios for decision-makers and the general public for informing climate actions. If we adopt a medium or lower emission scenario such as SSP245 or SSP126, it will not only limit the level of greenhouse warming but also permit sufficient incoming solar radiation, with wide-ranging implications for energy supply and ecosystem services.

## RESULTS AND DISCUSSION

### Estimated decline of surface incoming solar radiation in the historical period

**Figure 1.**
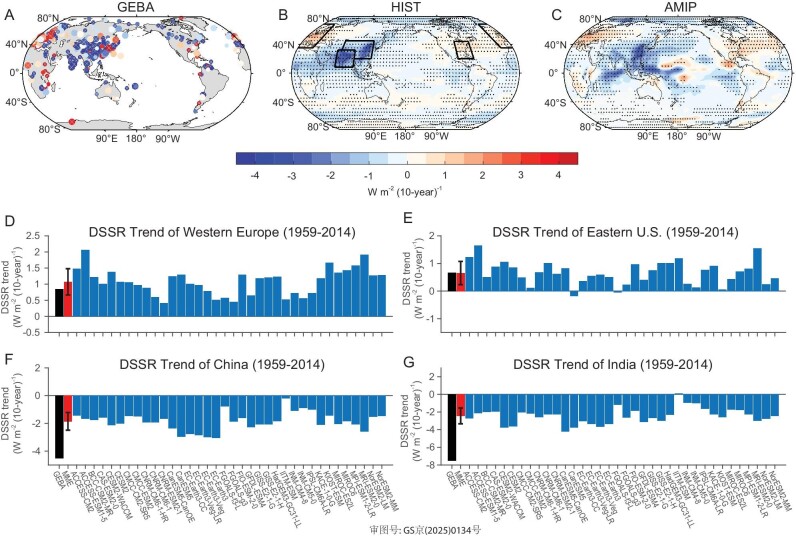
Historical DSSR trends in observations and simulations. Spatial pattern of linear trends of annual-mean DSSR during 1959–2014 in (A) GEBA, (B) multi-model mean of HIST and (C) AMIP runs from CMIP6 models. ‘×’ in (A) and dots in (B and C) indicate that the linear trends are significant at the 95% confidence level. The larger dots in (A) represent stations with recorded years >42 years, while the smaller points are between 28 years and 42 years. Linear trend of regional-average DSSR during 1959–2014 in (D) Western Europe (20°W–25°E, 35°–70°N), (E) Eastern U.S. (70°–95°W, 20°–44°N), (F) China (75°–125°E, 20°–45°N) and (G) India (68°–97°W, 8°–32°N) in GEBA (black), HIST runs (blue) and multi-model mean (red), respectively. Error bars are calculated as one standard deviation of DSSR trends among models. Due to the lack of spatial distribution of observing stations, we first gridded the space to 2.5 degrees × 2.5 degrees, averaged the DSSR trends recorded by stations within the grid point, and then compared them with the model output at that grid point.

To investigate changes of DSSR in the historical period, we first examine the spatial pattern of DSSR trends during 1959–2014 from the GEBA station datasets (Fig. 1A). Most evident features are the increase of DSSR in Europe and the eastern USA and the decrease of DSSR in the East and South Asia. These features are qualitatively well captured by the ensemble mean of CMIP6 models (Fig. 1B, C) in either the 36 historical (HIST) runs or the 72 AMIP (Atmospheric Model Intercomparison Project) runs. External historical forcings are applied in the HIST runs of fully coupled climate models, while external forcings and observed internally-driven SSTs are both applied in the AMIP models (see Materials and Methods). The strikingly similar patterns in both HIST and AMIP runs suggest that external forcings are the dominant drivers, while internal variability is of secondary importance. To quantify the model performance in the DSSR trend, a comparison between CMIP6 simulations and GEBA stations over some data-rich areas such as western Europe, eastern China, India, and the eastern USA was conducted (Fig. 1D–G). The observed decrease of DSSR over eastern China and India and the increase over western Europe and the eastern USA are captured in almost all CMIP6 models, although the magnitude is still underestimated in CMIP6 models in eastern China and India. Hence, it is justifiable to utilize CMIP6 models to assess historical changes of global DSSR and their causes, considering the global coverage of CMIP6 models.

**Figure 2.**
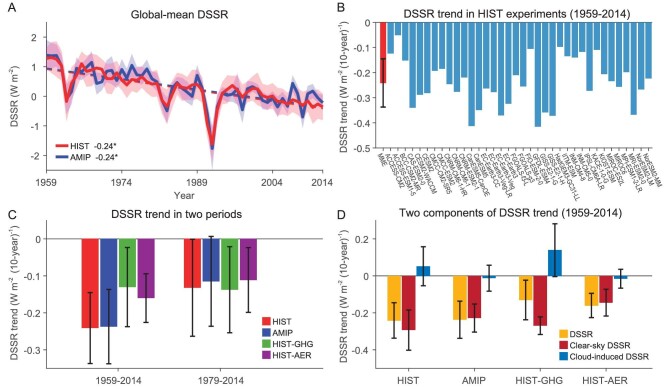
Historical changes of global mean DSSR and two components in simulations. (A) Time series of global mean DSSR in a 36-member ensemble mean of HIST runs (red) and ensemble average of 72 AMIP runs (blue) from CMIP6 models over the period 1959–2014. Shadings mark the one standard deviation model spread. The reference period is 1980–2009. Dashed lines are the linear fits and ‘*’ indicates the linear trends being significant at the 95% confidence level. (B) Linear trends of global mean DSSR during 1959–2014 in HIST runs (blue) and muti-model ensemble mean (MME; red). (C) Multi-model mean of linear trends for global mean DSSR during two periods (1959–2014 and 1979–2014) of HIST (red), AIMP (blue), HIST-GHG (green) and HIST-AER (purple) runs. (D) Multi-model mean of linear trends for global mean DSSR (yellow) and its two components (clear-sky, red; cloud-induced, blue) during 1959–2014 under HIST, HIST-GHG, HIST-AER and AMIP runs. Error bars on the multi-model mean are calculated as one standard deviation of DSSR trends among models.

Figure 2A shows the time evolution of global mean DSSR for the period 1959–2014 from HIST and AMIP runs. A clear downward trend in global DSSR has been consistently shown. The ensemble mean trend of the 36 CMIP6-HIST is −0.24 W m^−2^ (10y)^−1^. The decline trends are also evident in all the AMIP runs, with similar magnitudes. All the CMIP6 models show evident decreasing trends during 1959–2014 (Fig. 2B). The similar magnitudes of downward trends in both HIST and AMIP runs suggest that internal variability is not important for the period. Instead, external forcing dominates the historical global decline in DSSR. Based on separate experiments with individual forcings HIST-GHG and HIST-AER (see Materials and Methods), it is found that anthropogenic aerosol is an important driver of DSSR decline as previously suggested, but notably, GHGs also play an important role (Fig. 2C). Aerosol forcing is behind a sharp decline before the 1970s and the influence of GHGs has become important during the satellite period. Quantitatively, aerosols and GHGs contribute 66.4% and 54.1% to the global decline in DSSR during 1959–2014. However, during 1979–2014, the contribution from GHGs is larger than aerosols by 27.3%. This is consistent with the clean-air legislation in Europe and North America that has led to a reduction in aerosol emissions while GHG emissions have continued to rise in this period. It is also interesting to note that the decline rate of DSSR is slower during 1979–2014 than that during 1959–2014, which is consistent with previous findings of a surface brightening in some regions in the later period [8].

Changes in DSSR can be divided into two components, one is the change of clear-sky DSSR which is tied to water vapor and aerosols, and the other is the change of cloud-induced DSSR, defined as the difference between total DSSR and clear-sky DSSR (see Materials and Methods). Results from CMIP6 models suggest that the decline of solar radiation in the historical period is mainly caused by clear-sky solar radiation change, slightly offset by cloud-induced DSSR (Fig. 2D). The spatial patterns of clear-sky DSSR changes resemble DSSR changes in the HIST runs (Fig. 1B vs. Fig. S2A), further suggesting the dominant role of the clear-sky component in DSSR changes in the CMIP6 models. The main difference between total DSSR and clear-sky DSSR in the HIST experiments occurs in the midlatitudes, reflecting the role of clouds. Under both GHG and aerosol forcings, the clear-sky component dominates the simulated DSSR changes (Fig. 2D). Changes of cloud-induced DSSR have the same signs as the clear-sky DSSR changes under aerosol forcing, while the two are opposite under GHG forcing. The reduction in clear-sky DSSR under GHG forcing is horizontally even with a maximum along the equatorial region (Fig. S2B). Under aerosol forcing, there is a distinct spatial pattern with a large decrease in East and South Asia and an increase in the eastern USA and Europe (Fig. S2C). By comparing clear-sky DSSR changes between HIST-GHG and HIST-AER runs, one can clearly see that aerosol forcing is dominant in regions with large aerosol changes, while GHG forcing is more important over most oceans and high latitudes.

**Figure 3.**
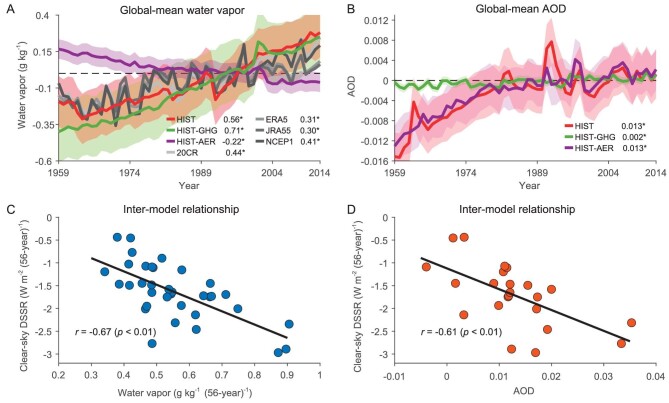
Key roles of water vapor and aerosol in the historical clear-sky DSSR changes. Time series of (A) global annual-mean water vapor and (B) global annual-mean AOD during 1959–2014. The reference period is 1980–2009. The grey, red, green and purple lines represent the corresponding global average, in re-analyses (water vapor in 20CR, ERA5, JRA-55 and NCEP1 and AOD in MERRA2), HIST, HIST-GHG and HIST-AER, respectively. The linear trends during 1959–2014 are given and ‘*’ represents the linear trends being significant at the 95% confidence level. Shadings represent one standard deviation of models. Scatter plots of global mean (C) water vapor and (D) AOD linear trends vs. global mean clear-sky DSSR (W m^−2^) linear trends (per 56-y) during 1959–2014 in HIST. Each dot represents one model. Correlation coefficients and *p* values are marked.

As the clear-sky component is the dominant term in DSSR changes in both all-forcings runs (HIST and AMIP) and individual forcing runs (HIST-GHG and HIST-AER), the time evolutions of two main factors influencing the clear-sky DSSR (water vapor and aerosols) are further examined in observations and model simulations (Fig. 3). Increasing GHG concentrations in the atmosphere drive up temperature. Water vapor content increases consequently following the Clausius–Clapeyron relationship. Warming is partly offset by aerosol forcing and so is water vapor (Fig. 3A). Aerosol concentrations, represented by aerosol optical depth (AOD), have globally increased substantially during 1959–2014 but kept almost unchanged during the satellite period (Fig. 3B). The constant AOD during the satellited period corresponds to a shift of aerosols from the western hemisphere to the eastern hemisphere as previously suggested [41,42], which is also well reproduced in CMIP6 models (Fig. S3). The increases of global mean water vapor content and AOD have both contributed to the decline of global mean clear-sky solar radiation (Fig. 3C, D). The correlation coefficients between the two terms and clear-sky DSSR changes in the HIST experiments are −0.67 and −0.61, respectively. Therefore, both the warming-induced increase of water vapor and the increase of aerosols are responsible and equally important for the global decline in clear-sky DSSR in the past decades.

### The competing roles of global warming and aerosols in the future changes of DSSR

**Figure 4.**
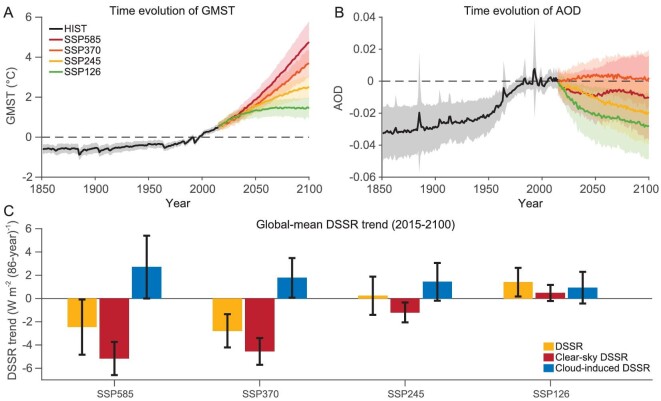
Contrasting future changes of global mean DSSR among four emission scenarios. Time series of (A) GMST and (B) AOD during 1850–2100 based on the CMIP6 MME. The reference period is 1980–2009. The black, red, light red, yellow and green lines represent HIST, SSP585, SSP370, SSP245 and SSP126 scenarios, respectively. Shading represents the multi-model spread calculated as one standard deviation of models. (C) Linear trends of global mean DSSR (yellow) and its two components (clear-sky DSSR (red) and cloud-induced DSSR (blue)) during 2015–2100 under the SSP585, SSP245 and SSP126 scenarios. Error bars are calculated as one standard deviation based on every model.

As shown above, in the historical period, increased water vapor and aerosol emissions work together to reduce DSSR. However, in the future, the two terms might work towards the opposite direction because aerosol emissions may come down due to air-pollution control while water vapor content continues to increase with ongoing global warming. To examine the impacts of both global warming and aerosol emission levels on future changes of DSSR, we use four different climate change scenarios: SSP126, SSP245, SSP370 and SSP585, with 35, 27, 33 and 32 models, respectively (see Materials and Methods). Global warming levels and aerosol concentrations are quite different in these four scenarios (Fig. 4A, B), allowing us to investigate the competing influences of these two factors. Under the SSP585 scenario (Fig. 4C) during 2015–2100, there is a 2.5 W m^−2^ decrease of DSSR globally, with a 1.2 W m^−2^ decrease over land and a 3.0 W m^−2^ decrease over the ocean (Fig. S4A). Regionally, the most evident increase occurs over East and South Asia, with the increase over eastern China reaching ∼16 W m^-2^, while the most evident decrease occurs over the equatorial Pacific and the Southern Ocean, which reaches −16 W m^−2^ (Fig. S4B). Under SSP370, the decrease of DSSR is even stronger than SSP585 (−2.8 W m^−2^ vs. −2.5 W m^−2^), albeit with less global warming, indicating a larger role played by aerosols (Fig. 4C). Under SSP245, the global mean DSSR keeps almost unchanged during 2015–2100 (only slightly increased by 0.2 W m^−2^; Fig. 4C), with a 1.7 W m^−2^ increase over land but a 0.4 W m^−2^ decrease over the ocean (Fig. S4E). Despite the fact we may see an ∼3 K warming under this scenario, the countering effects of rising temperature under global warming and decreasing aerosol emissions cancel each other out. Spatially, the linear trends of DSSR in SSP245 resemble those in SSP585, but with a larger increase over land and a weaker decrease over the ocean (Fig. S4F vs. Fig. S4B). Under SSP126, global DSSR would change into an increase of 1.4 W m^−2^ (Fig. 4C), with more contributions from land than ocean (2.8 vs. 0.8 W m^−2^; Fig. S4G). Albeit with global warming of ∼2 K under SSP126, the DSSR would be increased, suggesting the minor role of water vapor increase compared to the reduction in aerosols. The increase in DSSR occurs in almost all land areas, while an evident decrease can only be seen in the equatorial Pacific and the Southern Ocean (Fig. S4H).

As shown above, although GMST would significantly increase in all four scenarios, global mean DSSR shows contrasting changes across these scenarios, with significant decreases in SSP585 and SSP370, almost no changes in SSP245 and significant increases in SSP126. By decomposing the DSSR into clear-sky and cloud-induced components, it can be seen that there is a clear competition between clear-sky and cloud-induced DSSR changes in all scenarios except SSP126 (Fig. 4C). The clear-sky DSSR shows a significant decrease under SSP585 and SSP370, overwhelming the role of cloud-induced DSSR and leading to the overall decrease of DSSR. In the SSP245, the two components cancel each other out, resulting in almost no change of DSSR. In the SSP126, both the increases of clear-sky and cloud-induced DSSR are marginal, but their combinations lead to a robust increase of DSSR.

It is interesting to note that the cloud-induced DSSR increases under all scenarios. The magnitude of the increase seems to scale with the magnitude of GMST changes across different schemes. Indeed, there is a significant inter-model correlation between cloud-induced DSSR changes and GMST changes across scenarios, with 0.64, 0.52, 0.66 and 0.78 in the SSP585, SSP370, SSP245 and SSP126 scenarios, respectively. Why does global warming cause a cloud-induced DSSR increase? Since the positive cloud-induced DSSR changes are evident in all four scenarios, we use SSP585 with the highest cloud-induced DSSR changes as an example to investigate the physical mechanisms behind the cloud-induced DSSR changes. As shown in Fig. S5, the cloud-induced DSSR increase is closely tied to changes in cloud amount, in terms of both global mean (with an inter-model correlation of −0.73) and global distribution of trends (with a pattern correlation of −0.86). That means, under global warming, the decreased cloud amount would lead to increased incoming solar radiation. Consistent with cloud-induced DSSR changes, cloud amount is also highly correlated with GMST (Fig. S5E), with higher correlations in the subtropics and mid-latitudes (Fig. S6A). As the subtropics and mid-latitudes are mainly occupied by low clouds, enhanced atmospheric static stability under global warming would act to reduce the amount of low cloud [43–47]. It is interesting to note that the reduction in cloud amount is more evident over land than over ocean (−2.5% vs. −1.6%; Fig. S5B). This can be understood in terms of reduced relative humidity over land, with an inter-model correlation of −0.52 for the global land average and significant correlations in almost all land areas (Fig. S6B), which is also consistent with previous findings on the role of relative humidity in the cloud amount [44,46].

In contrast to future changes of cloud-induced DSSR, global mean clear-sky DSSR shows different changes across scenarios (Fig. S7), with a significant decrease in both SSP585 and SSP370 (−5.16 and −4.58 W m^−2^, respectively), a weak decrease in the SSP370 scenario (−1.20 W m^−2^) and a slight increase in the SSP126 scenario (0.47 W m^−2^). Spatially, South and East Asia see the most evident increase in clear-sky DSSR in all scenarios except SSP370, in which clear-sky DSSR only marginally increases over East Asia and even decreases over South Asia. As water vapor shows a similar dependence on the GMST changes across different scenarios (∼0.60 g kg^−1^ K^−1^ in all four), the different behaviors of clear-sky DSSR should be linked to different aerosol evolutions and global warming levels across scenarios (Fig. 4A, B). SSP245 and SSP126 would see the strongest reductions of aerosol emissions (Fig. 4B) but SSP585 and SSP370 would see the strongest global warming (Fig. 4A). By examining inter-model relationships with clear-sky DSSR, it is interesting to see higher correlations with AOD than GMST in the SSP245 and SSP126 scenarios (−0.85 and −0.78 for AOD vs. −0.43 and −0.44 for GMST, respectively). That is, clear-sky DSSR is more closely related to AOD in the SSP245 and SSP126. It is opposite in the SSP370 and SSP585. The contrast in the correlation coefficients reflects the relative importance of global warming and aerosols in clear-sky DSSR changes and its dependence on future climate change scenarios.

In summary, we have found two important factors determining changes of DSSR, i.e. global warming and aerosol concentrations. Global warming itself can have two competing effects on DSSR changes: on one hand, it increases DSSR by reducing the amount of cloud; on the other hand, it decreases DSSR by water vapor absorption as water vapor increases with rising temperature. The relative importance of these two effects is estimated using the HIST-GHG runs (see Materials and Methods). It is found that the net effects of global warming on DSSR are dominated by water vapor, as clear-sky DSSR has a much higher dependence on the GMST than cloud-induced DSSR (Fig. S8A, C). The aerosols-only simulations HIST-AER are used to estimate the dependence of clear-sky and cloud-induced DSSR on AOD. As aerosols can affect both temperature and DSSR at the same time, the temperature effect is first removed (see Materials and Methods). It is found that under aerosols-only forcing, the increased AOD leads both clear-sky and cloud-induced DSSR to work together to reduce DSSR, with clear-sky DSSR changes more than two times larger than cloud effect (Fig. S8B, D), and vice versa for the reduced AOD.

**Figure 5.**
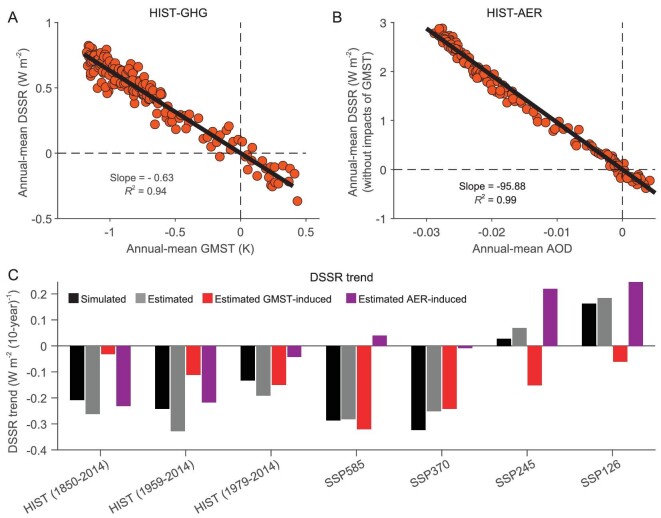
Predicting the past and future changes of DSSR by GMST and AOD. (A) Scatter plots of global annual mean surface temperature (GMST) changes vs. DSSR changes during 1850–2014 in HIST-GHG experiments. (B) Scatter plots of aerosol optical depth (AOD; dimensionless) changes vs. DSSR changes after removing the impacts of GMST during 1850–2014 in HIST-AER experiments. Each dot represents a year during 1850–2014 in the MME and the reference period is 1980–2009. (C) Linear trends of simulated DSSR (black), its estimates (grey) and two components (GMST-induced (red) and AER-induced (purple)) during 1850–2014, 1959–2014 and 1979–2014 under HIST and during 2015–2100 under the SSP585, SSP370, SSP245 and SSP126 scenarios.

Given the high dependence of both cloud-induced DSSR and clear-sky DSSR on the GMST and AOD, it is plausible to predict DSSR changes based on the inputs of GMST and AOD. A linear regression model is constructed (see Materials and Methods). The regression coefficient of DSSR with GMST is −0.63 W m^−2^ K^−1^ and AOD is −95.88 W m^−1^ based on the CMIP6 HIST-GHG and HIST-AER simulations (Fig. 5A, B). By examining changes of GMST and AOD over time, it is found that although GMST would increase significantly in all four scenarios (Fig. 4A), AOD would sharply decrease under both SSP126 and SSP245 scenarios (−2.20 × 10^−2^ for SSP126 and −1.96 × 10^−2^ for SSP245 during 2015–2100), but marginally decreased under the SSP585 scenario (only −3.51 × 10^−3^ during 2015–2100) and even slightly increased under SSP370 (Fig. 4B). By combining the regression coefficients obtained from Fig. 5A, B and the time evolutions of GMST and AOD from Fig. 4A, B, we can estimate past and future changes in DSSR. As shown in Fig. 5C, the simulated DSSR trend during 1850–2024 is −0.21 W m^−2^ (10-y)^−1^, and the estimated trend from our prediction model is −0.26 W m^−2^ (10-y)^-1^. The linear regression model also holds well at shorter historical periods (1959–2014 and 1979–2014). For future changes, CMIP6 simulated the DSSR trend for the period 2015–2100 to be −0.29 W m^−2^ (10-y)^−1^ under the SSP585 scenario, and the linear model predicted trend to be −0.28 W m^−2^ (10-y)^−1^, with a bias <2%. The prediction model works well for all the other scenarios. The difference between the estimated and simulated DSSR may be caused by the complex nonlinear interactions of water vapor, aerosols and clouds, which is not considered here.

During the whole historical period (1850–2014), aerosol forcing is dominant in changes of DSSR, but GMST becomes more important in recent decades, with a dominant role in the satellite period (1979–2014). For the future, GMST would dominate in the high radiative forcing scenarios (SSP585 and SSP370), while AOD would dominate in the low radiative forcing scenario (SSP126). For the moderate radiative forcing scenario SSP245, the effects of GMST and AOD almost cancel each other out, leading to weak changes of DSSR. Similarly, we can also well estimate the clear-sky and cloud-induced DSSR changes in the historical period and future scenarios based on the GMST and AOD in this regression model (Fig. S8E, F). Hence, by giving the GMST and AOD changes, which are tied to climate model sensitivity and future aerosol emission policy, the future changes of global DSSR and its two components can be well predicted.

## CONCLUSIONS

Here we show that DSSR has decreased globally based on CMIP6 model simulations in the past 6 decades mainly attributed to its clear-sky component, with a reduction of over 10% in some regions, which is in line with findings at widespread land-based observation stations. The decline of global mean DSSR has a comparable magnitude between CMIP6 AMIP runs and historical runs, suggesting the dominant role of external forcing. By separating the GHGs and aerosols based on GHG-only and aerosol-only simulations, it is found that GHGs and aerosols have comparable contributions to the past global DSSR changes, with more contributions from aerosols in the earlier period (1959–1978) but more contributions from GHG via increased water vapor in the satellite period (1979–2014).

In the future, whether the DSSR continues to decrease depends on both global warming levels and aerosol concentrations. Based on these two important factors, we constructed a linear regression model to predict past and future changes of DSSR with a high accuracy and the relative contributions of global warming and aerosols are quantified. In the whole historical period (1850–2014), aerosols play a dominant role in the DSSR changes, while global warming has become more important in recent decades and even overwhelms the role of aerosols during the satellite period (1979–2014). For future changes, in the low radiative forcing scenario SSP126, the sharp decrease of aerosol emissions plays a dominant role in driving the increase of DSSR, while the aerosol-induced increase of DSSR is almost canceled out by the global warming-induced decrease of DSSR in SSP245. In the high radiative forcing scenarios SSP585 and SSP370, global warming dominates the future decrease of DSSR. Hence, in the future with stronger global warming, we would see evident decreases of DSSR, increasing the difficulties in obtaining solar energy to replace fossil energy. Instead, when we adopt a stricter aerosol emission, such as SSP245 and SSP126, the DSSR could remain almost unchanged or even slightly increased, allowing a smoother transition from traditional fossil energy to a cleaner one.

## MATERIALS AND METHODS

### Observational and reanalysis datasets

In order to examine the historical evolution of the DSSR changes during 1959–2014 and better justify the CMIP6 model simulations, the DSSR from the GEBA station observations is used [39]. Because there are missing values in the GEBA data in some years, we calculated trends by considering only stations with recorded years of data more than half the length of the study period (i.e. ≥28 years). It is found that CMIP6 modes have well reproduced the observed DSSR trends in China, Europe and India, which have a relatively abundant number of stations (Fig. 1). The specific humidity from ERA5 [48], JRA-55 [49], 20CR [50] and NCEP1 [51] are used to examine the observed water vapor changes during 1959–2014. AOD from the Moderate Resolution Imaging Spectroradiometer (MODIS) Deep Blue retrieval [52] and the Modern-Era Retrospective analysis for Research and Applications, Version 2 (MERRA2) [53] are also used to examine the observed aerosol optical depth changes during 2001–2023 and 1980–2023, respectively.

### Model simulations

We use monthly solar radiation and related variables from CMIP6 model simulations [40] in the historical (termed as HIST) experiments from 1850 to 2014 and future scenarios from 2015 to 2100. Four future scenarios are considered, namely SSP585, SSP370, SSP245 and SSP126. The SSP585 scenario would have additional radiative forcing of 8.5 W m^−2^ by the year 2100, representing a future world in which the social and economic development is based on an intensified exploitation of fossil fuel resources but the air pollution is being tackled successfully. The SSP370 scenario would have additional radiative forcing of 7.0 W m^−2^ by the year 2100, representing a future world of regional rivalry in which the greenhouse gases emissions are always increasing by the year 2100 and some regional aerosol emissions would not be reduced much. The SSP245 would have additional radiative forcing of 4.5 W m^−2^ by the year 2100, representing a future world in which it adopts a medium pathway of future greenhouse gas emissions and climate protection measures are being taken. The SSP126 would have an additional radiative forcing of 2.6 W m^−2^ by the year 2100, representing a future world in which it adopts a sustainable and ‘green’ pathway and climate protection measures are being taken. See Ref. [54,55] for more details of these four scenarios. The historical, SSP585, SSP370, SSP245 and SSP126 simulations include 36, 35, 27, 33 and 32 models, respectively. We also utilize 72 members from 16 Atmospheric Model Intercomparison Project (AMIP) models from 1959 to 2014 to isolate the impacts of SST on DSSR. By comparing the historical and AMIP experiments, we can quantify the role of internal SST variability in the changes to DSSR. To quantify the relative roles of greenhouse gases (GHGs) and anthropogenic aerosols (AERs) in the past changes of DSSR, we also utilize the individual forcing simulations in the historical period (1850–2014) with 69 members from 13 CMIP6 models, which provide multiple members of GHG-only and AER-only simulations, termed as HIST-GHG and HIST-AER, respectively. Compared to GHG and AER, the influence of other external forcing, such as ozone, is found to be quite small by examining the corresponding individual forcing simulations.

To obtain the MME in the AMIP, HIST-GHG and HIST-AER runs, we first average the DSSR across different members in models providing more than one member, then average the DSSR across different models to ensure all models have the same weight. Note that there are fewer models conducting the HIST-GHG and HIST-AER experiments than the HIST experiment, so we re-calculate the contributions of GHG and AER to the past DSSR changes by using the same model sets to examine its influences on our conclusions. It is found that during 1959–2014, the contribution of aerosols and GHGs has been changed from 66.4% to 68.8% and 54.1% to 56.0%, respectively. Hence, our main conclusions are not affected much by these model number differences. Detailed information of specific model simulations is described in Table S1.

For comparing the absorption of solar radiation by water vapor between CMIP5 and CMIP6 models, we use HIST-GHG experiments of CMIP5 and CMIP6 from 10 corresponding model centers. The regression coefficient of clear-sky DSSR onto surface water vapor is adopted to quantify the ability of each model to absorb solar radiation. As shown in Fig. S1, the underestimation of solar radiation absorption by water vapor has been greatly alleviated in CMIP6 (MME: −2.17 W m^−2^ g^−1^ kg) compared with CMIP5 (MME: −1.50 W m^−2^ g^−1^ kg). The members of HIST-GHG experiments of CMIP5 are as follows: ACCESS1-3 (3), bcc-csm-1–1 (1), CanESM2 (5), CESM1-CAM5 (1), CNRM-CM5 (5), HadGEM2-ES (3), IPSL-CM5A-LR (4), MIROC-ESM (3), MRI-CGCM3 (1) and NorESM1-M (1), for 27 members in total.

### Prediction of clear-sky DSSR based on GMST and AOD

The global mean DSSR, and its two components clear-sky DSSR and cloud-induced DSSR are closely tied to the GMST and global mean AOD, so we construct a linear regression model based on GMST and global mean AOD to predict them. Following is an example of the prediction of DSSR:


(1) \begin{eqnarray*}
\textrm{DSSR} = a*\textrm{GMST} + b*\textrm{AOD} + \textrm{other}\,\,\textrm{processes}
\end{eqnarray*}

(2) \begin{eqnarray*}
\textrm{HIST}_{{\rm GHG}}:\,\,\textrm{DSSR} = a*\textrm{GMST} + \textrm{other}\,\,\textrm{processes}
\end{eqnarray*}

(3) \begin{eqnarray*}
&&\textrm{HIST}_{{\rm AER}}:\,\,\textrm{DSSR} - a*\textrm{GMST}\\
&&\quad = b*\textrm{AOD} + \textrm{other}\,\,\textrm{processes}
\end{eqnarray*}

To determine the parameter *a*, we use the linear regression of global mean DSSR onto GMST in the HIST-GHG, as in this experiment, the changes in the DSSR are mainly determined by water vapor, which is almost linearly scaled to the GMST. To determine the parameter *b*, we use the linear regression of global mean DSSR after removing the impacts of GMST onto AOD in the HIST-AER, as in this experiment, the changes in the DSSR are mainly determined by both GMST and global mean AOD. After obtaining *a* and *b*, we estimate the DSSR in the future scenarios by taking GMST and AOD from corresponding scenarios. These relationships are found to also hold for clear-sky DSSR and cloud-induced DSSR.

## Supplementary Material

nwaf007_Supplemental_File

## Data Availability

CMIP6 model data is available at https://esgf-index1.ceda.ac.uk/search/cmip6-ceda/. ERA5 data is available at https://cds.climate.copernicus.eu/cdsapp#!/dataset/reanalysis-era5-single-levels. JRA-55 data is available at https://rda.ucar.edu. NCEP1 data is available at https://psl.noaa.gov/data/gridded/data.ncep.reanalysis.html. 20CR data is available at https://psl.noaa.gov/data/20thC_Rean/. MERRA-2 data is available at https://gmao.gsfc.nasa.gov/reanalysis/MERRA-2/data_access/. The GEBA data is available at https://geba.ethz.ch/. The Level 3 monthly average MODIS AOD data are from https://ladsweb.modaps.eosdis.nasa.gov.

## CODE AVAILABILITY

The codes to generate the figures and table are based on MATLAB R2022b and will be available from the corresponding authors upon request.
