# Supplementary material for: A long-term decline in downward surface solar radiation
Source: Natl Sci Rev. 2025 Jan 25;12(3):nwaf007. doi: 10.1093/nsr/nwaf007 (PMC11846084; doi:10.1093/nsr/nwaf007)
Supplement: nwaf007_Supplemental_File [file nwaf007_supplemental_file.docx]

# **Supplementary materials:**

# **A long-term decline in downward surface solar radiation**

# Fengfei Song^*1,2^, Yudi Mao^1,3^, Shichu Liu^1,3,4^, Lixin Wu^*1,2^, Lu Dong^1,2^, Hui Su^5^, Yawen Wang^3^, Boriana Chtirkova^6^, Peili Wu^7^, Martin Wild^6^

1. Frontier Science Center for Deep Ocean Multispheres and Earth System and Physical Oceanography Laboratory, Ocean University of China, Qingdao 266100, China,

2. Laoshan Laboratory, Qingdao 266237, China

3. College of Oceanic and Atmospheric Sciences, Ocean University of China, Qingdao 266100, China

4. Key Laboratory of Physical Oceanography and Frontiers Science Center for Deep Ocean Multispheres and Earth System/Academy of the Future Ocean, Ocean University of China, Qingdao 266100, China

5. Department of Civil and Environmental Engineering, The Hong Kong University of Science and Technology, Hong Kong 999077, China

6. ETH Zurich, Institute for Atmospheric and Climate Science, Zurich 8092, Switzerland

7. Met Office Hadley Centre, Exeter, EX1 3PB, UK

*** Corresponding authors:**

Fengfei Song (songfengfei@ouc.edu.cn) and Lixin Wu ([lxwu@ouc.edu.cn](mailto:lxwu@ouc.edu.cn))

There are 1 table and 8 figures in this appendix.


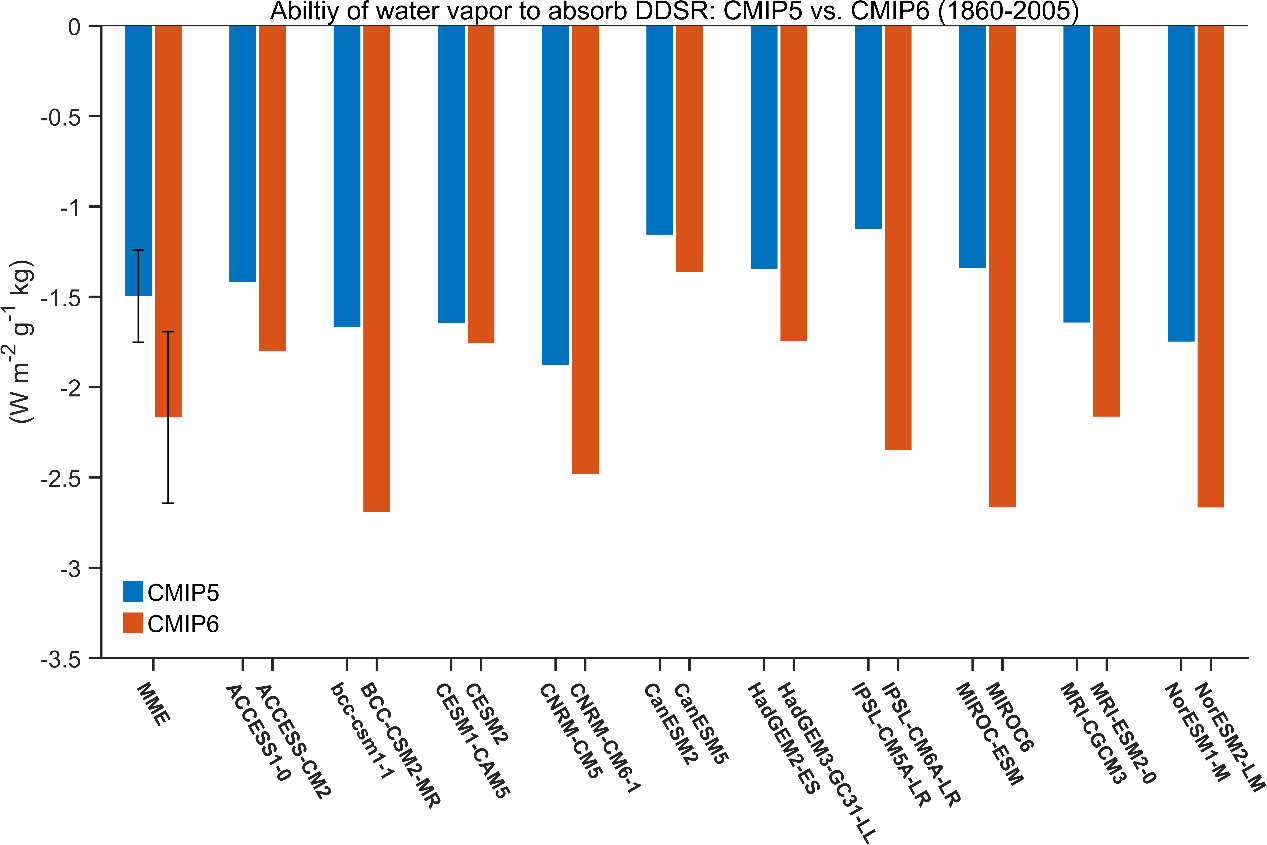


**Fig. S1 Improved ability of shortwave absorption by water vapor in CMIP6 models compared to CMIP5 models.** Regression coefficient of annual-mean clear-sky DSSR onto annual-mean surface water vapor during 1860-2005 in 5^th^ (CMIP5; blue) and 6^th^ (CMIP6; red) generation of 10 CMIP models in the GHG-only experiment. Error bars on the multi-model mean are calculated as one standard deviation among models.

**
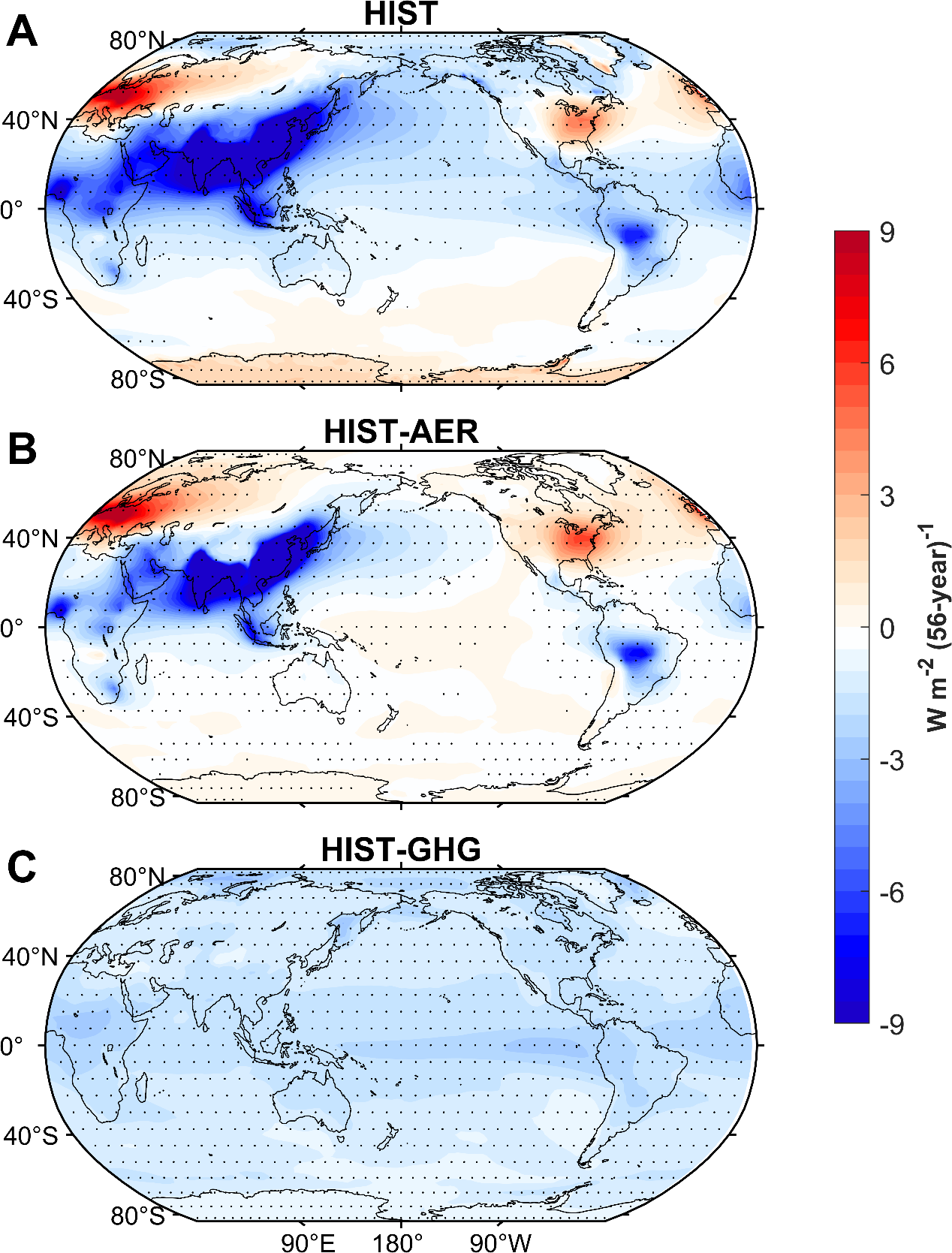
**

**Fig. S2 Historical changes of global clear-sky DSSR in simulations.** Spatial pattern of linear trends of annual-mean clear-sky DSSR (unit: W m^−2^ (56-y)^−1^) during 1959-2014 in (**A**) HIST, (**B**) HIST-AER and (**C**) HIST-GHG. Dots indicate that the linear trends are significant at the 95% confidence level.


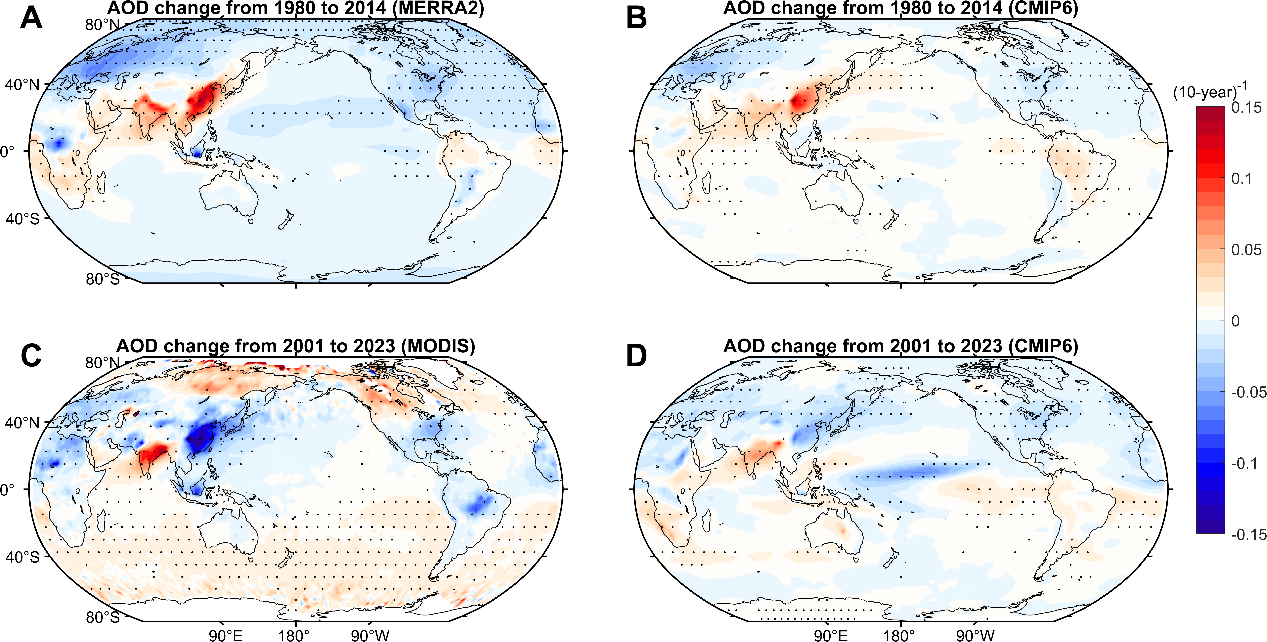


**Fig. S3** Linear trends of AOD during 1980-2014 in (**A**) MERRA2 and (**B**) multi-model ensemble mean of historical simulations from 32 CMIP6 models. The dots indicate the trend is significant at 95% confidence level. (**C**-**D**) is the same as (**A**-**B**) but for (**C**) MODIS and (**D**) multi-model ensemble mean of historical (2001-2014) plus SSP585 scenario (2015-2023) from 25 CMIP6 models during 2001-2023.


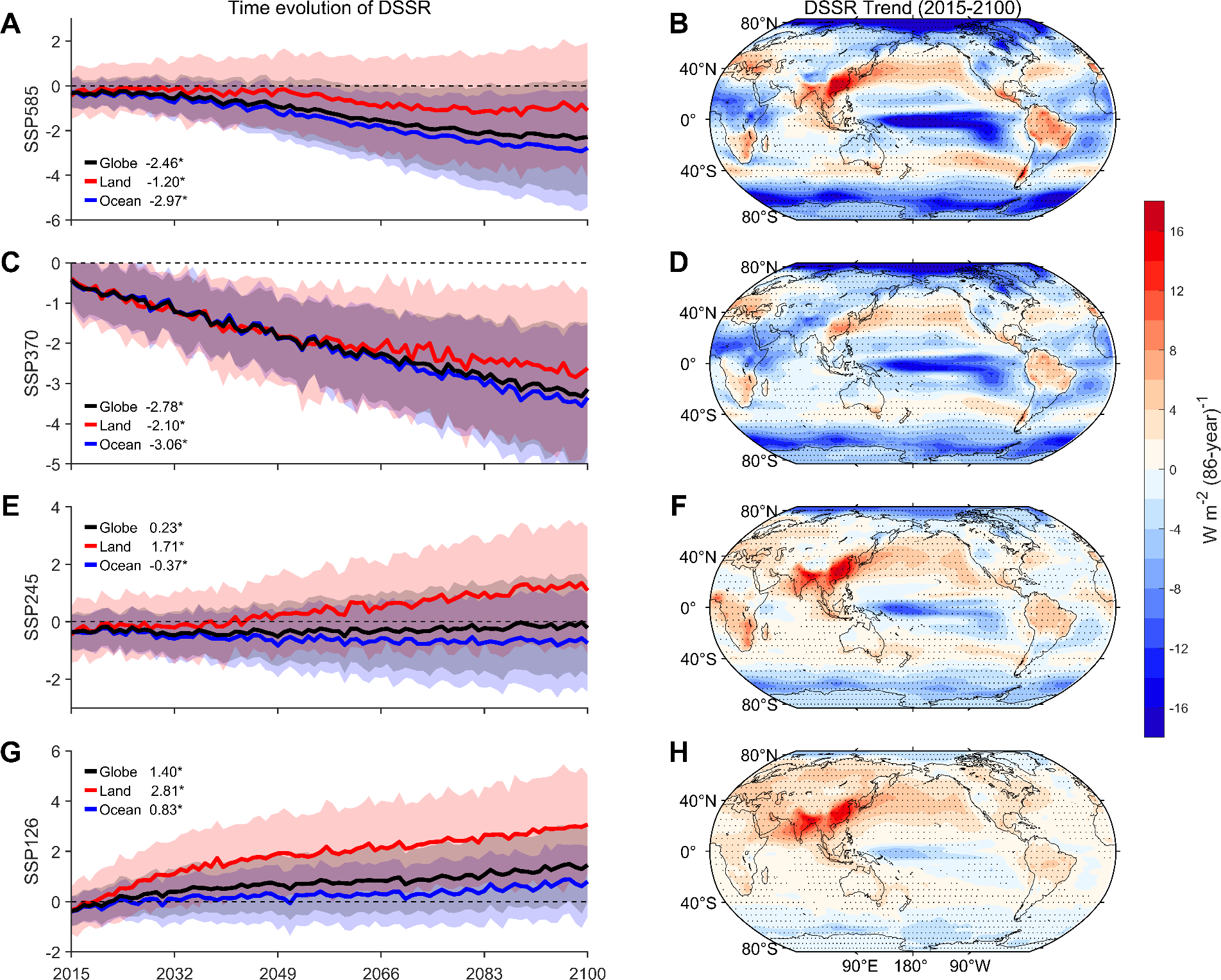


**Fig. S4 Future changes in DSSR among four emission scenarios.** Time series of annual-mean DSSR anomalies in (**A**) SSP585, (**C**) SSP370, (**E**) SSP245 and (**G**) SSP126 during 2015-2100. The reference period is 1980-2009 based on the historical experiments. Black, red and blue lines represent the average over the globe, land and ocean, respectively and the linear trends (unit: W m^−2^ (86-y)^−1^) during 2015-2100 are given, where “*” indicates that the trends are significant at the 95% confidence level. Shading represents the multi-model spread calculated as one standard deviation of models. Spatial pattern of linear trends of annual-mean DSSR (unit: W m^−2^ (86-y)^−1^) during 2015-2100 in (**B**) SSP585, (**D**) SSP370, (**F**) SSP245 and (**H**) SSP126. Dots indicate that the linear trends are significant at the 95% confidence level.

**
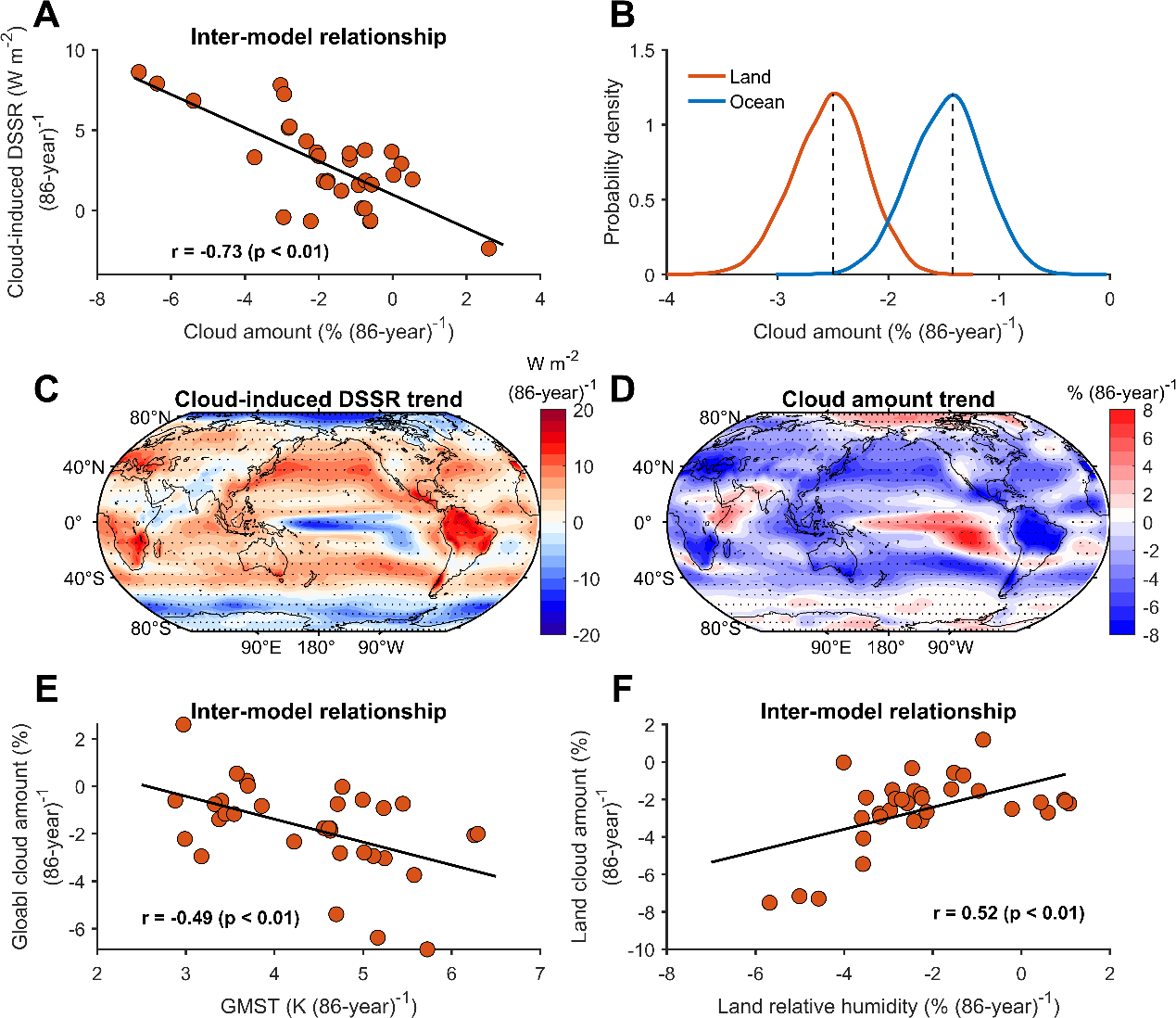
**

**Fig. S5 Future changes of cloud amount and its impact on the DSSR in SSP585.** (**A**) Inter-model relationship between global-mean cloud amount linear trend (unit: % (86-y)^−1^) and global-mean cloud-induced DSSR linear trend (unit: W m^−2^ (86-y)^−1^) during 2015-2100 in SSP585. Each dot represents one model. The correlation coefficient is −0.73, significant at the 99% confidence level. (**B**) Probability density function of 10,000 realizations of a bootstrap method for changes in cloud amount (units: % (86-y)^−1^) of land (red) and ocean (blue) in the SSP585 scenario during 2015-2100 based on 33 CMIP6 models. Spatial pattern of linear trends of (**C**) cloud-induced DSSR and (**D**) cloud amount during 2015-2100 based on CMIP6 multi-model ensemble mean. The dots indicate the regions where the regression coefficient is significant at the 95% confidence level. (**E**) Inter-model relationship between ocean cloud amount linear trend and GMST linear trend (unit: K (86-y)^−1^) during 2015-2100 under SSP585. (**F**) Inter-model relationship between land cloud amount linear trends and land relative humidity linear trends (unit: % (86-y)^−1^) during 2015-2100 under SSP585. The correlation coefficient is −0.49 and 0.52, respectively, both significant at the 99% confidence level.

**
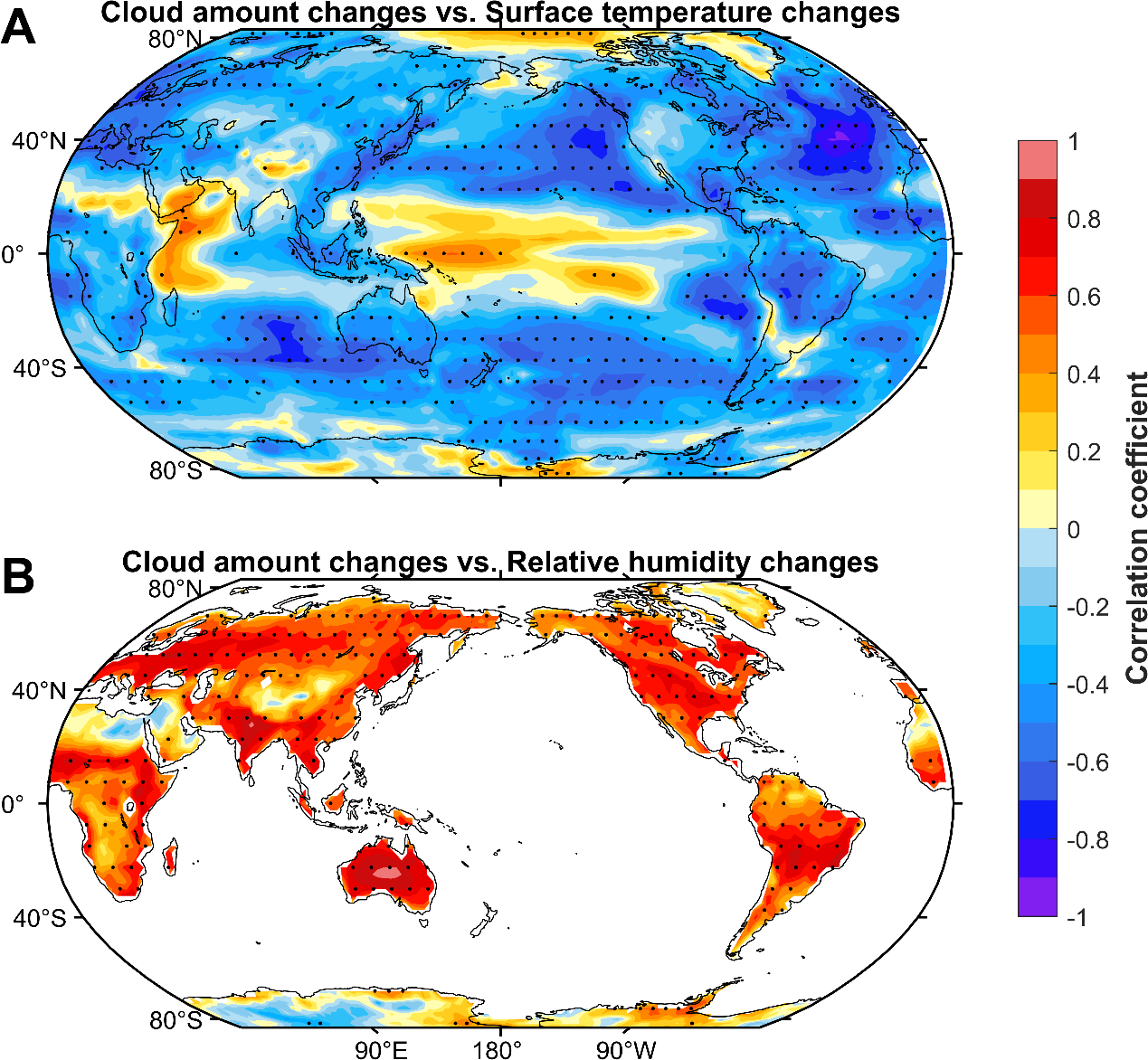
**

**Fig. S6 Relative humidity and surface temperature play key roles in the future land and ocean cloud amount changes, respectively.** The spatial pattern of inter-model correlation between (**A**) cloud amount linear trends (unit: % (86-y)^−1^) vs. and surface temperature linear trends (unit: K (86-y)^−1^) (**B**) cloud amount linear trends vs. relative humidity linear trends (unit: % (86-y)^−1^) during 2015-2100 in SSP585 among CMIP6 models. Dots indicate the correlations are significant at the 95% confidence level.

**
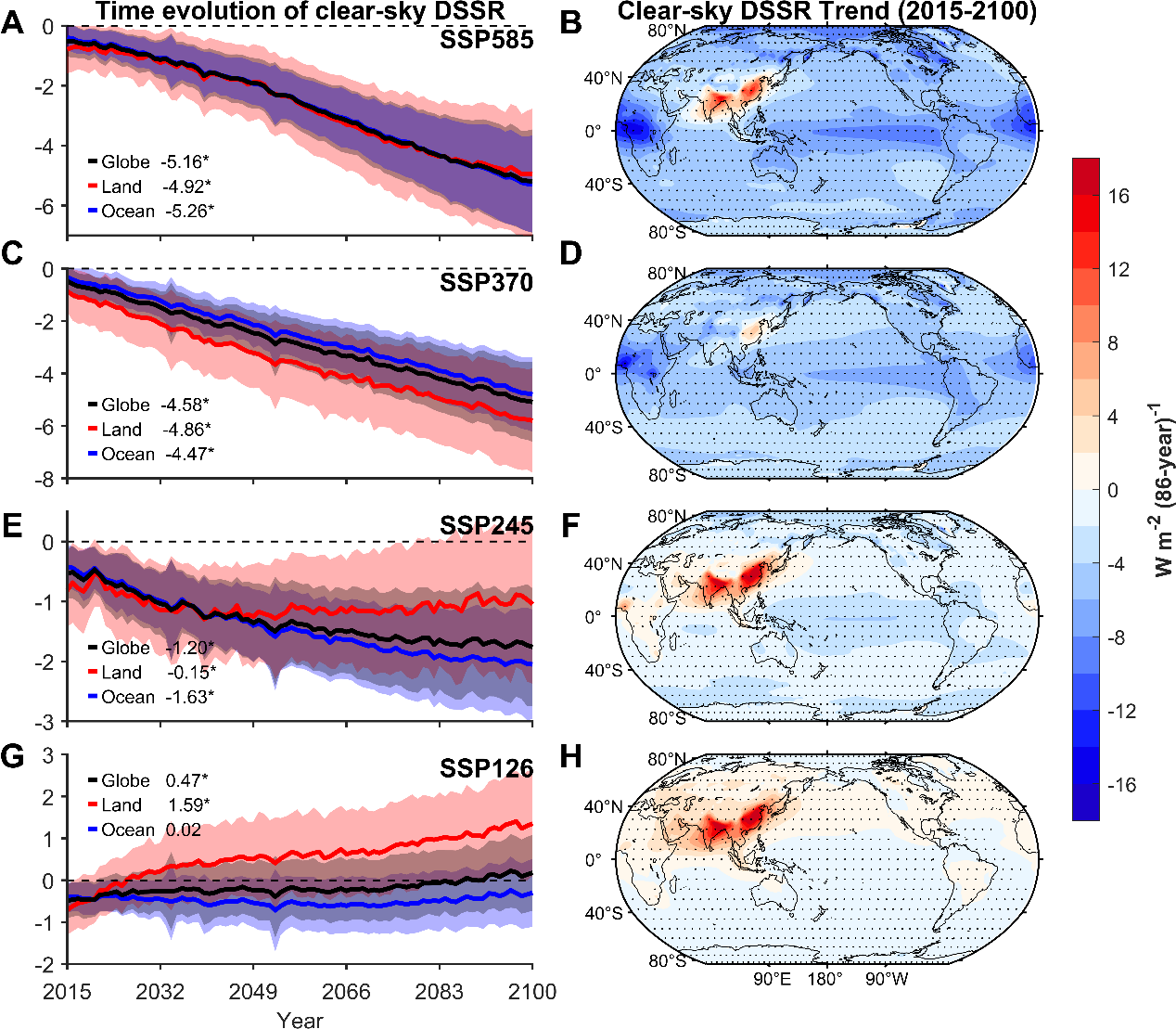
**

**Fig. S7 The contrasting future changes of clear-sky DSSR between four emission scenarios.** Time series of annual-mean clear-sky DSSR anomaly (unit: W m^−2^) in (**A**) SSP585, (**C**) SSP370, (**E**) SSP245 and (**G**) SSP126 during 2015-2100. The reference period is 1980-2009. Black, red and blue lines represent the average over the globe, land and ocean, respectively. Linear trends during 2015-2100 are given (unit: W m^−2^ (86-y)^−1^) and “*” represents the trends are significant at the 95% confidence level. Shading represents the ±1 standard deviation of models. Spatial pattern of linear trends of annual-mean clear-sky DSSR (unit: W m^−2^ (86-y)^−1^) during 2015-2100 in (**B**) SSP585, (**D**) SSP370, (**F**) SSP245 and (**H**) SSP126. Dots indicate that the linear trends are significant at the 95% confidence level.


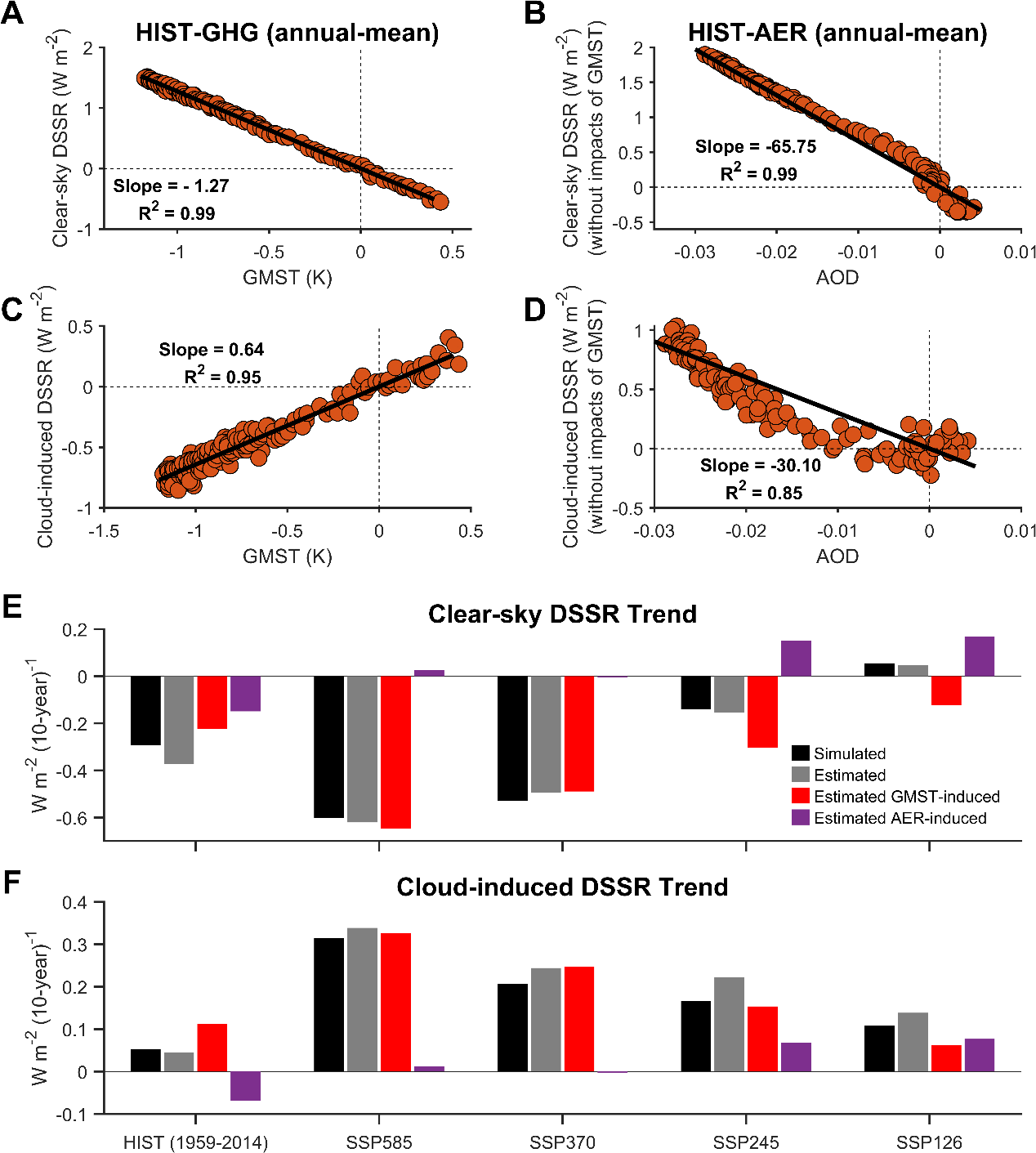


**Fig. S8 GMST and AOD are used to predict the past and future changes of clear-sky DSSR and cloud-induced DSSR.** (**A**) Scatter plots of global annual mean surface temperature (GMST; unit: K) changes vs. clear-sky DSSR (unit: W m^−2^) changes during 1850-2014 in HIST-GHG scenarios. (**B**) Scatter plots of aerosol optical depth (AOD; dimensionless) changes vs. clear-sky DSSR changes after removing the impacts of GMST during 1850-2014 in HIST-AER scenarios. Each dot represents one year and the reference period is 1980-2009. (**C**), (**D**) As in (**A**), (**B**) respectively, but for cloud-induced DSSR. (**E**) Linear trends of simulated clear-sky DSSR (black), its estimates (grey) and two components (GMST-induced (red) and AER-induced (purple)) during 1959-2014 under HIST and during 2015-2100 under the SSP585, SSP245 and SSP126 scenarios. Units: W m^−2^ (10y)^−1^. (**F**) As in (**E**), but for cloud-induced DSSR.

**Table S1 | The CMIP6 model variables used in this study.**

| CMIP6 Model | DSSR/Clear-sky DSSR | Cloud amount | Specific humidity | 550nm Aerosol Optical Depth | Relative humidity | Surface Temperature |
| --- | --- | --- | --- | --- | --- | --- |
| ACCESS-CM2 | H, S1, S2, S3, S5, HG(3), HA(3) | H, S5, HG(3), HA(3) | H, S1, S2, S3, S5, HG(3), HA(3) | H, S1, S2, S3, S5,HG(3), HA(3) | S5 | H, S1, S2, S3, S5 |
| ACCESS-ESM1-5 | H, S1, S2, S3, S5, HG(3), HA(3) | H, S5, HG(3), HA(3) | H, S1, S2, S3, S5, HG(3), HA(3) | H, S1, S2, S3, S5, HG(3), HA(3) | S5 | H, S1, S2, S5 HG(3), HA(3) |
| BCC-CSM2-MR | H, A(1), S3, S5, HG(3), HA(3) | HG(3), HA(3) | H, S3, S5, HG(3), HA(3) | H |  | H, S3, S5, HG(3) |
| CanESM5 | H, A(10), S1, S2, S3, S5, HG(1), HA(1) | H, S5, HG(1), HA(1) | H, S1, S2, S5, HG(1), HA(1) | H, S1, S2, S5, HG(1), HA(1) | S5 | H, S1, S2, S5, HG(1), HA(1) |
| CanESM5-CanOE | H, S1, S2, S3, S5 | H, S5 | H, S1, S2, S3, S5 | H, S1, S2, S3, S5 | S5 | H, S1, S2, S3, S5 |
| CAS-ESM2-0 | H, S1, S2, S3, S5 | H, S5 | H, S1, S2, S5 |  | S5 | H, S1, S2, S3, S5 |
| CESM2 | H, A(3), S1, S2, S5, HG(1), HA(1) | H, S5, HG(1), HA(1) | H, S1, S2, S3, S5, HG(1), HA(1) | H, S1, S2, S5, HG(1), HA(1) | S5 | H, S1, S2, S3, S5, HG(1), HA(1) |
| CESM2-WACCM | H, S1, S2, S3, S5 | H, S5 | H, S1, S2, S3, S5 | H, S1, S2, S5 | S5 | H, S1, S2, S3, S5 |
| CIESM | A(3) |  |  |  |  |  |
| CMCC-CM2-SR5 | H, S1, S2, S3, S5 | H, S5 | H, S1, S2, S3, S5 | H, S1, S2, S3, S5 | S5 | H, S1, S2, S3, S5 |
| CMCC-ESM2 | H, S1, S2, S3, S5 | H, S5 | H, S1, S2, S3, S5 | H, S1, S2, S3, S5 | S5 | H, S1, S2, S3, S5 |
| CNRM-CM6-1 | H, A(10), S1, S2, S3, S5, HG(10), HA(10) | H, S5, HG(10), HA(10) | H, S1, S2, S3, S5, HA(10) | H, S1, S2, S3, S5, HA(3) | S5 | H, S1, S2, S3, S5, HG(10), HA(10) |
| CNRM-CM6-1-HR | H, A(1), S1, S2, S3, S5 | H, S5 | H, S1, S2, S3, S5 | H, S1, S2, S3, S5 | S5 | H, S1, S2, S3, S5 |
| CNRM-ESM2-1 | H, A(1), S1, S2, S3, S5 | H, S5 | H, S1, S2, S3, S5 | H, S1, S2, S3, S5 | S5 | H, S1, S2, S3, S5 |
| E3SM-2-0 | HG(5), HA(5) | HG(5), HA(5) | HG(5), HA(5) | HG(5), HA(5) |  | HG(5), HA(5) |
| EC-Earth3 | H, S1, S2, S3, S5 | H, S5 | H, S1, S2, S3, S5 | H, S1, S2, S3, S5 | S5 | H, S1, S2, S3, S5 |
| EC-Earth3-CC | H, S2, S5 | H, S5 | H, S2, S5 |  | S5 | H, S2, S5 |
| EC-Earth3-Veg | H, S1, S2, S3, S5 | H, S5 | H, S1, S2, S3, S5 | H, S1, S2, S3, S5 | S5 | H, S1, S2, S3, S5 |
| EC-Earth3-Veg-LR | H, S1, S2, S3, S5 | H, S5 | H, S1, S2, S3, S5 |  | S5 | H, S1, S2, S3, S5 |
| FGOALS-f3-L | H, A(3), S1, S2, S3, S5 | H, S5 | H, S1, S2, S3, S5 |  | S5 | H, S1, S2, S3, S5 |
| FGOALS-g3 | H, A(3), S1, S2, S3, S5 | H, S5 | H, S1, S2, S3, S5 |  | S5 | H, S1, S2, S3, S5 |
| FIO-ESM-2-0 | H, S1, S2, S5 | H, S5 | H, S1, S2, S5 |  | S5 | H, S1, S2, S5 |
| GFDL-ESM4 | H, S1, S2, S3, S5 | H, S5 | H, S1, S2, S3, S5 | H, S1, S2, S3, S5 | S5 | H, S1, S2, S3, S5 |
| GISS-E2-1-G | H, S1, S2, S3, S5, HG(10), HA(10) | H, S5, HG(10), HA(10) | H, S1, S2, S3, S5, HG(10), HA(10) |  | S5 | H, S1, S2, S3, S5, HG(10), HA(5) |
| GISS-E2-1-H | H, S5 |  |  |  |  | H, S5 |
| HadGEM3-GC31-LL | H, S1, S2, S5, HG(5), HA(5) | H, S5, HG(5), HA(5) | H, S1, S2, S5, HG(5), HA(5) | H, S1, S2, S5, HG(5), HA(5) | S5 | H, S1, S2, S5, HG(5), HA(5) |
| IITM-ESM | H, A(1) | H, S5 | H |  | S5 | H |
| INM-CM4-8 | H, S1, S2, S3, S5 | H, S5 | H, S1, S2, S3, S5 | H, S1, S2, S3, S5 | S5 | H, S1, S2, S3, S5 |
| INM-CM5-0 | H, S1, S2, S3, S5 | H, S5 | H, S1, S2, S3, S5 | H, S1, S2, S3, S5 | S5 | H, S1, S2, S3, S5 |
| IPSL-CM6A-LR | H, A(20), S1, S2, S3, S5, HG(10), HA(10) | H, S5, HG(10), HA(10) | H, S1, S2, S3, HG(10), HA(10) | H, S1, S2, S3, S5, HG(10), HA(10) | S5 | H, S1, S2, S3, S5, HG(10), HA(10) |
| KACE-1-0-G | H, S1, S2, S5 | H, S5 | H, S1, S2, S5 | H, S1, S2, S5 | S5 | H, S1, S2, S5 |
| KIOST-ESM | H, S1, S2, S5 | H, S5 | H, S1, S2, S5 |  | S5 | H, S1, S2, S5 |
| MIROC6 | H, A(5), S1, S2, S5, HG(10), HA(10) | H, S5, HG(10), HA(10) | H, S1, S2, S5, HG(10), HA(10) | H, S1, S2, S5, HG(3), HA(3) | S5 | H, S1, S2, S5, HG(10), HA(10) |
| MIROC-ES2L | H, S1, S2, S3, S5 | H, S5 | H, S1, S2, S3, S5 | H, S1, S2, S3, S5 | S5 | H, S1, S2, S3, S5 |
| MPI-ESM1-2-LR | H, S1, S2, S3, S5 | H, S5 | H, S1, S2, S3, S5 | H, S1, S2, S3, S5 | S5 | H, S1, S2, S3, S5 |
| MRI-ESM2-0 | H, A(5), S1, S2, S3, S5, HG(5), HA(5) | H, S5, HG(5), HA(5) | H, S1, S2, S3, S5, HG(5), HA(5) | H, S1, S2, S3, S5, HG(5), HA(5) | S5 | H, S1, S2, S3, S5, HG(5), HA(5) |
| NorESM2-LM | H, S1, S2, S3, S5, HG(3), HA(3) | H, S5, HG(3), HA(3) | H, S1, S2, S3, S5, HG(3), HA(3) | H, S1, S2, S3, S5, HG(3), HA(2) | S5 | H, S1, S2, S3, S5, HG(3), HA(3) |
| NorESM2-MM | H, S1, S2, S3, S5 | H, S5 | H, S1, S2, S3, S5 | H, S1, S3, S5 | S5 | H, S1, S2, S3, S5 |
| TaiESM1 | A(3) |  |  |  |  |  |
| CAMS-CSM1-0 | A(3) only for DSSR |  |  |  |  |  |

“H” denotes the historical simulation; “A” denotes the AMIP simulations; “S1”, “S2”, “S3” and “S5” denote the SSP1-2.6, SSP3-7.0, SSP2-4.5, SSP5-8.5 simulations, respectively; “HG”, “HA” denote the GHGs and AERs simulations, respectively. The numbers in brackets represent the number of ensemble members in the CMIP6 simulations when it is greater than 1.
